# Next-Generation Sequencing Identifies Novel *PMPCA* Variants in Patients with Late-Onset Dominant Optic Atrophy

**DOI:** 10.3390/genes13071202

**Published:** 2022-07-05

**Authors:** Majida Charif, Arnaud Chevrollier, Naïg Gueguen, Selma Kane, Céline Bris, David Goudenège, Valerie Desquiret-Dumas, Isabelle Meunier, Fanny Mochel, Luc Jeanjean, Fanny Varenne, Vincent Procaccio, Pascal Reynier, Dominique Bonneau, Patrizia Amati-Bonneau, Guy Lenaers

**Affiliations:** 1 MitoLab Team, UMR CNRS 6015-INSERM U1083, Institut MitoVasc, SFR ICAT, Université d’Angers, 49933 Angers, France; ma.charif@ump.ac.ma (M.C.); arnaud.chevrollier@univ-angers.fr (A.C.); nagueguen@chu-angers.fr (N.G.); selmakane@hotmail.com (S.K.); cebris@chu-angers.fr (C.B.); david.goudenege@chu-angers.fr (D.G.); vadesquiret@chu-angers.fr (V.D.-D.); viprocaccio@chu-angers.fr (V.P.); pareynier@chu-angers.fr (P.R.); dobonneau@chu-angers.fr (D.B.); pabonneau@chu-angers.fr (P.A.-B.); 2 Genetics and Immuno-Cell Therapy Team, Mohammed First University, Oujda 60000, Morocco; 3 Departments of Biochemistry and Molecular Biology, University Hospital Angers, 49933 Angers, France; 4 National Reference Centre for Inherited Sensory Diseases, University Hospital of Montpellier, University of Montpellier, 34000 Montpellier, France; isabelannemeunier@yahoo.fr; 5 Institut des Neurosciences de Montpellier, INSERM U1051, Université de Montpellier, 34000 Montpellier, France; 6 Department of Genetics, AP-HP, Pitié-Salpêtrière University Hospital, 75013 Paris, France; fanny.mochel@upmc.fr; 7 Department of Ophthalmology, Nîmes University Hospital, CEDEX 9, 30900 Nîmes, France; luc.jeanjean@chu-nimes.fr; 8 Department of Ophthalmology, Hôpital Pierre Paul Riquet CHU Purpan, 31300 Toulouse, France; varenne.f@chu-toulouse.fr; 9 Departments of Genetics, University Hospital Angers, 49933 Angers, France; 10 Service de Neurologie, University Hospital Angers, 49933 Angers, France

**Keywords:** dominant optic atrophy, mitochondrial peptidase, mitochondrial dynamic, heterozygous variants, retinal ganglion cell degeneration

## Abstract

Dominant Optic Atrophy (DOA) is one of the most common inherited mitochondrial diseases, leading to blindness. It is caused by the chronic degeneration of the retinal ganglion cells (RGCs) and their axons forming the optic nerve. Until now, DOA has been mainly associated with genes encoding proteins involved in mitochondrial network dynamics. Using next-generation and exome sequencing, we identified for the first time heterozygous *PMPCA* variants having a causative role in the pathology of late-onset primary DOA in five patients. *PMPCA* encodes an α subunit of the mitochondrial peptidase (MPP), responsible for the cleavage and maturation of the mitochondrial precursor proteins imported from the cytoplasm into mitochondria. Recently, *PMPCA* has been identified as the gene responsible for Autosomal Recessive Cerebellar Ataxia type 2 (SCAR2) and another severe recessive mitochondrial disease. In this study, four *PMPCA* variants were identified, two are frameshifts (c.309delA and c.820delG) classified as pathogenic and two are missenses (c.1363G>A and c.1547G>A) classified with uncertain pathological significance. Functional assays on patients’ fibroblasts show a hyperconnection of the mitochondrial network and revealed that frameshift variants reduced α-MPP levels, while not significantly affecting the respiratory machinery. These results suggest that alterations in mitochondrial peptidase function can affect the fusion-fission balance, a key element in maintaining the physiology of retinal ganglion cells, and consequently lead to their progressive degeneration.

## 1. Introduction

Dominant optic atrophy (DOA, MIM *605290) is an inherited optic neuropathy with a prevalence estimated at 1 in 25,000 [1,2,3,4]. DOA affects visual acuity by altering the central visual field and color vision, due to a progressive loss of retinal ganglion cells (RGCs) and their axons that form the optic nerve, ensuring the transmission of visual information from the retina to the brain. Most patients have an age of onset in the first decade of life, some may experience functional blindness and others can be relatively asymptomatic [5]. DOA can be also a part of a syndromic condition called DOA*plus* in 20% of cases, with secondary symptoms affecting auditory, neuronal and muscular functions. Sixty to seventy per cent of DOA cases harbor pathogenic variants in the nuclear *OPA1* gene [6], which was the first gene identified to cause DOA [7,8]. But recently, an increasing number of novel DOA genes were identified through the introduction of next-generation sequencing technologies [9], including *OPA3*, *MFN2*, *SPG7*, *AFG3L2*, *DNM1L* and *SSBP1* [10,11,12,13,14,15,16,17]. These genes are all involved directly in mitochondrial function, mostly mitochondrial dynamics, with the exception of *SSBP1*, which is involved in mitochondrial DNA (mtDNA) replication. In addition, the *WFS1* gene, responsible for Wolfram syndrome, is also responsible for DOA associated with neuro-sensorial deafness [18] and for isolated recessive isolated optic atrophy [19].

In mitochondria, the mitochondrial processing peptidase (MPP) plays the most important role in preprotein processing compared to other mitochondrial peptidases [20]. *PMPCA* (MIM * 613036), a nuclear gene localized on human chromosome 9q34.3, encodes the a-subunit of mitochondrial processing peptidase (a-MPP), a protein that participates in the cleavage of the mitochondrial targeting peptide of nuclear-encoded mitochondrial precursor proteins upon their import into mitochondria [21]. Without its function, abnormal nuclear-encoded mitochondrial precursor proteins accumulate inside mitochondria, disrupt mitochondrial functions and halt cell growth. In 2015, *PMPCA* has been identified as the gene responsible for Autosomal Recessive Cerebellar Ataxia type 2 (SCAR2), a severe mitochondrial disease and later for a Leigh-like syndrome with spastic ataxia [22,23,24,25].

In the present study, we identified for the first time, heterozygous variants of *PMPCA* in five families with primary DOA. Fibroblasts characterization shows a tendency to mitochondrial network hyperconnection in *PMPCA* patient fibroblasts and revealed decreased levels of PMPCA protein.

## 2. Materials and Methods

### 2.1. Consent for Genetic Investigations

Written informed consent to perform genetic analyses was obtained from each subject involved in this study, according to protocols approved by the Ethical Committees of the different Institutes involved in this study, and in agreement with the Declaration of Helsinki (Institutional Review Board Committee of the University Hospital of Angers, Authorization number: AC-2012-1507).

### 2.2. Genetic Analysis

Genomic DNA was extracted from peripheral blood cells from cohorts of DOA and sporadic cases of optic atrophy initially screened for *OPA1*, *OPA3* and *WFS1* exonic sequences and all pathogenic variants in the mitochondrial DNA responsible for Leber hereditary optic neuropathy (LHON). Negative cases were analyzed using a resequencing gene panel dedicated to the clinical molecular diagnosis of inherited optic neuropathies (ION). Further negative samples, among which was the first patient with a *PMPCA* variant (family 3), were analyzed using whole-exome sequencing. Then *PMPCA* molecular screening was included in the ION panel and led to the discovery of families 1, 2, 4 and 5. Library preparation, sequencing, bioinformatics and variants analysis were carried out as previously described [10,12].

### 2.3. Fibroblasts Study

Fibroblasts from *PMPCA* individuals P1: II:1 and P2: II:2 from families 1 and 3 respectively, were generated from skin biopsies and cultured in 2/3 Dulbecco’s Minimum Essential Medium (DMEM, Gibco) supplemented with 1/3 AmnioMAX (Gibco, Thermo Fisher, Waltham, MA, USA), 10% fetal calf serum (Lonza, Portsmouth, NH, USA) and 1% Penicillin-Streptomycin-Amphotericin B (Lonza). Respiratory chain enzymatic activities and western blots were assessed as described [12,25], using the following antibodies: PMPCA (Novus Biologicals, CO, USA, #NBP1-89126; 56kD), PMPCB (Novus Biologicals, #NBP1-92120; 56kD); OPA1 (Abcam, Cambridge, England, ab42364; 95 and 85kD); Citrate Synthase (Abcam ab96600; 52kD).

### 2.4. Time Lapse and Deconvolution Microscopy

To assess the mitochondrial network dynamic [26], cells were incubated for 20 min with MitoTracker Green FM (Invitrogen™, Thermo Fisher, Waltham, MA, USA) to visualize mitochondria (green). Coverslips were mounted in a housing and placed on the stage of an inverted wide-field microscope ECLIPSE Ti-E (Nikon, Tokyo, Japan) equipped with a 100× oil immersion objective (Nikon Plan Apo100×, Tokyo, Japan, N.A. 1.45) and a NEO sCOMS camera controlled by Metamorph7.7 software (Molecular Devices, Sunnyvale, CA, USA). A precision, piezoelectric driver mounted underneath the objective lens allowed faster *Z*-step movements, keeping the sample immobile while shifting the objective lens. 35-one image planes were acquired along the *Z*-axis at 0.1 mm increments. For mitochondrial network characterization, acquired images were iteratively deconvolved using Huygens Essential 14.06 version software (Scientific Volume Imaging, Hilversum, The Netherlands), with a maximum iteration score of 50 and a quality threshold of 0.01. Imaris 8.0 ^®^ software (Oxford Instruments Brand Bitplane AG, Zurich, Switzerland) was used for 3D processing and morphometric analysis. Time-lapse images of 90 ms duration (5-sec interval) were acquired at a fixed temperature of 25 °C.

#### 2.4.1. Immunofluorescence

Human skin fibroblasts were seeded at a density of ~90,000 cells per well in a six-well plate containing 20-mm coverslips and incubated overnight. Cells were fixed with 4% paraformaldehyde (PFA) in PBS for 15 min. After fixation, cells were quickly washed 3 times in PBS and then incubated in the blocking buffer (BF; PBS with 5% BSA) for 15 min. Coverslips were then washed in PBS three times and incubated overnight with primary antibody diluted in the BF at 4 °C on a rocking platform providing a gentle “wave” effect. Coverslips were then washed in BF three times for 5 min and subsequently incubated for 90 min at room temperature with goat anti-mouse Alexa 647 IgG (H+L) secondary antibody diluted in BF in a dark chamber. Finally, coverslips were washed in PBS twicefor 5 min and kept in PBS at 4 degrees up to the assembly for the STORM acquisition.

Immunodetection of nucleoids was achieved on fixed fibroblasts using antibodies against DNA (clone AC-30-10) Progen GmbH and co-stained with Citrate synthase antibodies. Image treatment was performed using Imaris spot detection software (Imaris 8.0 ^®^) to visualize and quantify mtDNA/nucleoid number per cell and mtDNA/nucleoid spacing within mitochondria. For mitophagy exploration, co-staining with autophagosome marker anti-microtubule-associated protein 1 light chain 3α (LC3, ab48394, 1:500; Abcam) and mitochondrial marker citrate synthase was performed. Images acquisitions were performed using deconvolution microscopy.

#### 2.4.2. STORM Acquisition

For super-resolution imaging, the cavity of a clean single depression slide (Paul Marienfeld, Lauda-Königshofen, Germany) was filled with 50 μL of switching buffer (Abbelight, Paris, France) and covered by the coverslip, the sample side facing downward. The device was placed on the stage of an inverted motorized microscope NIKON ECLIPSE Ti-E (Nikon Instruments Europe, Amsterdam, The Netherlands) equipped with a CFI SR APO TIRF 100X ON1.49 objective, a Perfect Focus System and a total internal reflection fluorescence (TIRF) ILas2 module (Roper Scientific, Martinsried, Germany).

Acquisition of images was performed using Metamorph 7.7 software (Molecular Devices, CA, USA). Image sequences were acquired with a single-photon sensitive camera Evolve 128TM EMCCD 512 × 512 imaging array, 16 × 16 μm pixels (Photometrics, Tucson, AZ, USA). Acquisitions were performed at a fixed temperature of 25 °C in a dark heating chamber (Okolab NA, Pozzuoli, Italy). Phase contrast was first used for orientation and focus adjustment. Prior to STORM imaging, a multichannel TIRF fluorescence microscopy image was acquired for subsequent comparison with the STORM image. Images were acquired with an integration time of 60 ms per frame. The total acquisition time points for each sequence were adapted to the observed structure and to the labelling density (5000 to 20,000 frames). Images were analyzed and reconstructed using the WaveTracer module integrated into Metamorph software [27].

## 3. Results

### 3.1. Identification of PMPCA Variants in Individuals with Primary Dominant Optic Atrophy

**Figure 1:**
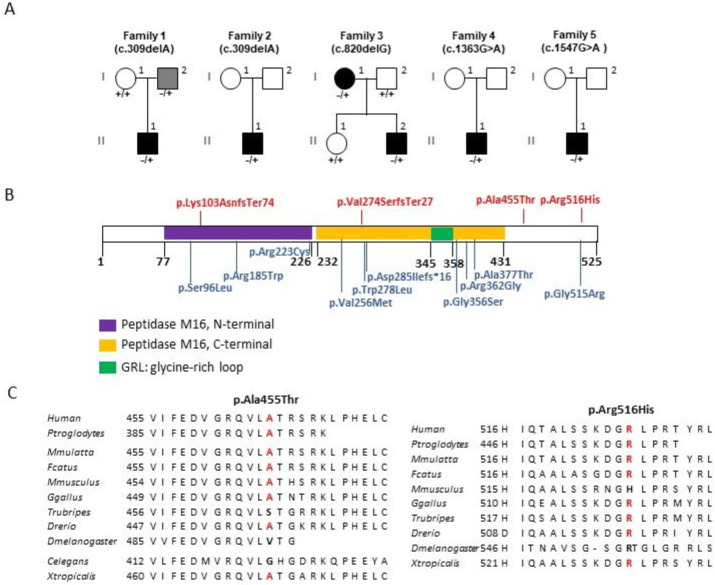
***PMPCA* pedigrees and amino acid change localization.** (**A**): Description of the pedigrees with *PMPCA* (NM_015160.3) variants and their segregation among the families. (**B**): The structure of the PMPCA protein with the amino acid changes associated with DOA on the top, and associated with Spinocerebellar ataxia, autosomal recessive 2 (SCAR2) and a progressive mitochondrial encephalopathy, on the bottom. (**C**): Evolutionary conservation of the p.Ala455 and p.Arg516 residues (in red) and their neighbor amino acids in eukaryotic PMPCA protein sequences of metazoans and invertebrates.

Using whole-exome sequencing, we screened for variants in unrelated patients presenting inherited optic neuropathies (ION) with a negative molecular diagnosis after analyzing *OPA1*, *OPA3*, *WFS1*, *SPG7*, *AFG3L2*, *DNM1L*, *MFN2* exonic sequences, and pathogenic variants in the mitochondrial DNA responsible for LHON. After eliminating frequent (>1/10.000) and non-pathogenic variants, according to the Sift, Polyphen and Mutation-Taster prediction tools, a first heterozygous pathogenic variant was identified in *PMPCA*, which led to the inclusion of this gene in the ION panel. Three additional heterozygous *PMPCA* (NM_015160.3) variants were identified in four families (Figure 1A), two unreported and two being referenced with an allele frequency of 4.01 × 10^−6^ and 8.01 × 10^−6^ (Figure 1B, Table 1). Two variants (c.309delA and c.820delG) are pair base deletions causing a frameshift (p.(Lys103AsnfsTer74) and p.(Val274SerfsTer27)); and two are missense variants (c.1363G>A and c.1547G>A) causing the p.(Ala455Thr) and p.(Arg516His) amino-acid changes, respectively (Table 1), which are evolutionary well conserved throughout animal PMPCA sequences (Figure 1C). These variants were confirmed by Sanger sequencing and analyzed for segregation, whenever DNA samples were available (Figure 1A). The pathogenicity of the two missense *PMPCA* variants was assessed in silico using multiple prediction software, with results strongly supporting their deleterious and damaging effect (Table 2), although they were classified as class 3 variants by the ACMG prediction program (Table 1).

### 3.2. Clinical Manifestations of PMPCA Patients

All individuals included were referred to ophthalmology departments due to poor visual acuity. All patients were males aged between 30 and 69 years, who were primarily diagnosed with isolated dominant optic atrophy; three had normal brain MRI imaging. Patients II.1 from family 1 and II.1 from family 4 disclosed severe optic atrophy with visual acuities ranging from 0.5/10 to counting fingers (Table 1). Additional associations or problems were observed: multiple sclerosis for patient II.1 from family 4 and peripheral neuropathy for patient II.1 from family 2 (Table 1).

### 3.3. Functional Effects of PMPCA Variants

**Figure 2:**
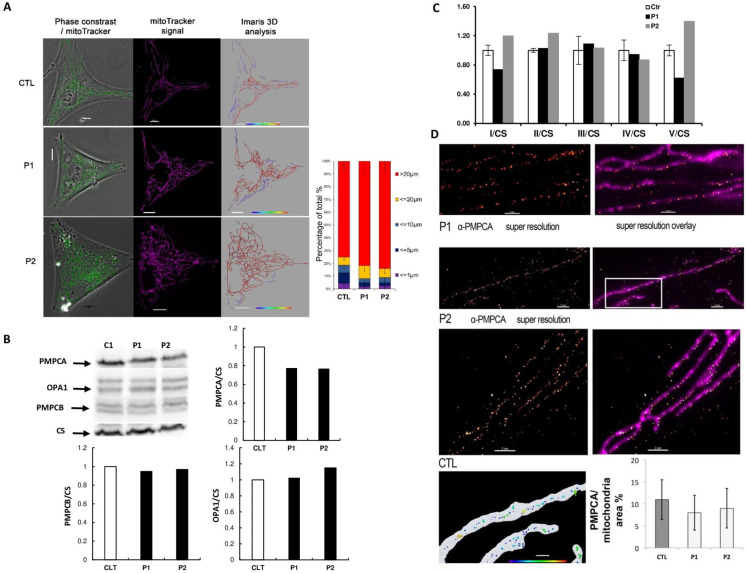
Mitochondrial dynamic and PMPCA distribution studies of fibroblasts from control and *PMPCA* mutated patients. (**A**): Representative fluorescent images of mitochondrial network structure overlaid on phase-contrast (on the left) showed a mitochondrial network hyperconnection in PMPCA fibroblasts. Mitochondrial volume (in purple on black background in the middle) was assessed using the Mitotracker Green fluorescent signal by Imaris software and color-coded on the right. The inset illustrates the classification code. To present the changes in mitochondrial morphology in patients’ cells, types of mitochondria were classified into 5 groups according to mitochondrial length: blobs < 1 μm; fragmented < 5 μm; tubular < 10 μm; filamentous < 20 μm; mitochondrial network > 20 um. Bar graphs show the distribution of the mitochondrial population of Control, P1 and P2. Mean ± SEM. Scale bar: 10 μm. (**B**): Western blots (left) against PMPCA, PMPCB, OPA1 and citrate synthase (CS) on control (C1) and two patients’ (P1 and P2) fibroblasts reveal decreased levels of PMPCA and equal levels of PMPCB and OPA1 in the pathological conditions, as shown on the histogram (right). (**C**): Enzymatic activities of the respiratory complexes (CI to CV) from the control and the PMPCA mutated fibroblast strains related to the citrate synthase (CS) enzymatic activity, did not reveal a significant difference between control and mutated fibroblasts. Biochemical data were generated using the two-tailed paired *t*-test. Results are Mean ± S.E.M. from four independent experiments. (**D**): Single-molecule localization microscopy dSTORM was used to analyze PMPCA distribution, correlated to total internal reflection fluorescence TIRF microscopy for mitochondrial staining. Using Imaris 8.0 ^®^ software, the dSTORM PMPCA immunofluorescence signal was used to quantify their mitochondrial surface protein distribution.

To evaluate the consequences of *PMPCA* variants on mitochondrial physiology, two fibroblast cell lines were derived from individuals P1: II:1 and P2: II:2 from families 1 and 3. Mitochondrial network overlaid on phase-contrast revealed hyperconnected structures in cells from *PMPCA* patients, compared to control (Figure 2A). Western blots experiments, using PMPCA, PMPCB, OPA1 and citrate synthase (CS) antibodies revealed a 25% reduction in PMPCA level, lower than expected for frameshift variants, and equal levels of PMPCB and OPA1 proteins (Figure 2B). Enzymatic activities of the respiratory chain complexes did not reveal a significant difference between control and mutated fibroblasts (Figure 2C). In addition, no alteration was noticed in the analysis of PMPCA distribution in the mitochondrial network (Figure 2D).

## 4. Discussion

Dominant optic atrophy is a heterogeneous group of diseases caused by selective loss of retinal ganglion cells (RGCs) and ascending degeneration of the optic nerve. In this study, we identified four heterozygous variants in *PMPCA* as a cause of DOA in five unrelated families. These variants are either novel or referenced with a very low frequency in the gnomAD database. The two missense variants are classified by the ACMG guidelines as variants of uncertain significance, although in silico analysis using the most common tools shows that these variants are probably damaging (Table 2) and were evolutionary well conserved in different animal species (Figure 1C). However functional studies are necessary to ascertain their full contribution to the disease.

Recessive pathogenic variants in *PMPCA* have been described in the context of non-progressive cerebellar ataxia and severe Leigh-like syndrome associated with spastic ataxia [22,23,24,25]. Thus, except for *OPA1*, all other DOA genes were initially identified as causing severe, essentially recessive, mitochondrial diseases, while dominant variants in these genes are causing DOA. Again, this is the case for *PMPCA*, for which we disclosed the involvement of heterozygous variants in a rather late-onset optic atrophy. Because, of the four variants identified, two were frameshifts classified as likely pathogenic, it should be advised to examine the parents of SCAR2 patients who are carriers of other frameshift variants. A similar situation has been observed for the *SPG7* gene, with the initial identification of rather mild optic atrophy in recessive *SPG7* patients with spastic paraplegia, followed later by the identification of dominant SPG7 variants in patients with restricted Dominant Optic Atrophy [10].

Interestingly, of the five patients identified with *PMPCA* variants, two had additional neurological symptoms, such as peripheral neuropathy for the index case of family 2 and multiple sclerosis for the index case from family 4. This is supported by previous observations that multisystemic manifestations, including neurological symptoms, are reported in up to 20% of *OPA1* pathogenic variant carriers and in LHON patients [28,29,30,31,32]. Thus, functional alterations of PMPCA might predispose patients to the emergence of neurological symptoms, in addition to jeopardizing RGC function and survival.

*PMPCA* encodes the α subunit of the mitochondrial processing peptidase (MPP), which cleaves the targeting peptide of nuclear-encoded mitochondrial precursor proteins upon their import into mitochondria [21]. Our observation of *PMPCA* fibroblasts revealed a decreased level of PMPCA protein, although not reaching a 50% decrease, as would be expected for cells with a frameshift variant and deeply contrasting with the observation of a severe PMPCA depletion in SCAR2 patient lymphoblasts, associated with impaired frataxin production and processing [23,25]. In addition, a hyperconnected mitochondrial network was observed in PMPCA conditions, as already reported in fibroblasts from DNM1L and OPA3 patients affected by DOA [12,15]. Conversely, we did not disclose the alteration of the respiratory chain, nor the PMPCA distribution in the mitochondrial network, but some rare events at mitochondria-autophagosome contact sites are suggestive of increased mitophagy (data not shown).

In summary, we suggest heterozygous variants in *PMPCA* gene as new candidates for DOA. We also present a novel pathophysiological mechanism responsible for retinal ganglion cell degeneration, most likely milder than the one responsible for SCAR2 presentation, caused by bi-allelic *PMPCA* pathogenic variants.

## 5. Conclusions

In conclusion, we report the first DOA patients with heterozygous *PMPCA* variants, confirming the genetic heterogeneity of autosomal inherited optic neuropathies and the important role of mitochondrial import in the maintenance of retinal ganglion cell integrity.

## Figures and Tables

**Table 1:** Clinical and Molecular Data of the *PMPCA* Patients.

Family	Patient	Sex	Age	VA	Other Symptoms	Brain MRI	ORF Change	Protein Change	rs #	Gnomad Freq.	ACMG Classification
1	(II.1)	M	30	counting fingers	-	normal	c.309delA	p.(Lys103AsnfsTer74)	unknown	-	PVS1 and PM2 Class 5
2	(II.1)	M	53		peripheral neuropathy	normal					
3	(II.2)	M	45	4/10	-	normal	c.820delG	p.(Val274SerfsTer27)	rs777445198	4.01 × 10^−6^	PVS1 and PM2 Class 5
4	(II.1)	M	35	0.5/10	multiple sclerosis	ND	c.1363G>A	p.(Ala455Thr)	unknown	-	PM2 and BP4 Class 3
5	(II.1)	M	69	6/10	-	ND	c.1547G>A	p.(Arg516His)	rs768196711	8.01 × 10^−6^	PM2 Class 3

Abbreviations: VA: visual acuity; M: male; rs #: reference sequence number; gnomAD Freq.: Frequency in the Genome Aggregation Database; ACMG: American College of Medical Genetics and Genomics PVS1: Null variant (nonsense, frameshift, canonical ±1 or 2 splice sites, initiation codon, single or multiexon deletion) in a gene where LOF is a known mechanism of disease. (Pathogenic, Very Strong). PM2: Absent from controls (or at extremely low frequency if recessive) in Exome Sequencing Project, 1000 Genomes Project, or Exome Aggregation Consortium. (Pathogenic, Moderate). BP4: Multiple lines of computational evidence suggest no impact on gene or gene product (conservation, evolutionary, splicing impact, etc.) (Benign, Supporting).

**Table 2:** In Silico Analysis of *PMPCA* Missense Variants.

Variant	Polyphen	SIFT	MutationTaster	FATHMM-MKL	LRT	PROVEAN	DANN
c.1363G>A (p.Ala455Thr)	0.568 possibly damaging	0.492 tolerated	0.9999 disease-causing	0.9334 damaging	0 deleterious	−2.46, −2.42, −2.78 damaging	0.9956 damaging
c.1547G>A (p.Arg516His)	0.017 benign	0.025 damaging	0.9999 disease-causing	0.9669 damaging	9.9999 × 10^−7^ neutral	−2.83, −2.53 damaging	0.9982 damaging
